# Acceptability of a Colorectal Cancer-Preventive Diet Promoting Red Meat Reduction and Increased Fiber and Micronutrient Intake: A Cross-Sectional Study in Romanian Adults

**DOI:** 10.3390/nu17142386

**Published:** 2025-07-21

**Authors:** Marius-Cătălin Belean, Teodor-Andrei Maghiar, Anca-Maria Căpraru, Andreea-Adriana Neamțu, Dan Iliescu, Valentin-Cristian Iovin, Flaviu-Ionuț Faur, Meda-Ada Bugi, Alina Totorean, Sorina Tăban, Sorin Dema, Cristina-Adriana Dehelean, Bogdan Dan Totolici, Ovidiu Laurian Pop, Octavian Crețu, Carmen Neamțu

**Affiliations:** 1 Doctoral School of Medicine, “Vasile Goldis” Western University of Arad, Liviu Rebreanu Str., No. 86, 310045 Arad, Romania; mariusbelean@yahoo.com (M.-C.B.); flaviu.faur@umft.ro (F.-I.F.); 2 Department of Surgery I, Clinical County Emergency Hospital of Arad, Andrenyi Karoly Str., No. 2–4, 310037 Arad, Romania; totolici.bogdan@uvvg.ro (B.D.T.); neamtu.carmen@uvvg.ro (C.N.); 3 Doctoral School of Biomedical Sciences, University of Oradea, Universității Str., No. 1, 410087 Oradea, Romania; teodormaghiar@yahoo.com; 4 Faculty of Medicine, University of Oradea, Universității Str., No. 1, 410087 Oradea, Romania; drovipop@yahoo.com; 5 Department of Surgery, Pelican Hospital, Corneliu Coposu Str., No. 2, 410450 Oradea, Romania; 6 Department of Pharmacy, Poiana Mare Psychiatry Hospital, Gării Str., No. 40, 207470 Poiana Mare, Romania; mitran.anca@hotmail.com; 7 Faculty of Pharmacy, “Vasile Goldis” Western University of Arad, Liviu Rebreanu Str., No. 86, 310045 Arad, Romania; bugi.ada@umft.ro; 8 Department of Toxicology, “Victor Babes” University of Medicine and Pharmacy, Eftimie Murgu Square, No. 2, 300041 Timisoara, Romania; cadehelean@umft.ro; 9 Research Centre for Pharmaco-Toxicological Evaluation, “Victor Babes” University of Medicine and Pharmacy, Eftimie Murgu Square, No. 2, 300041 Timisoara, Romania; 10 Department of Pathology, Clinical County Emergency Hospital of Arad, Andrenyi Karoly Str., No. 2–4, 310037 Arad, Romania; 11 Department of Pathology, “Pius Brinzeu” Clinical County Emergency Hospital Timisoara, Liviu Rebreanu Boulevard, No. 156, 300723 Timisoara, Romania; sorinataban@yahoo.com; 12 Department of Surgery I—Clinic of Surgical Semiotics & Thoracic Surgery, Center for Hepato-Biliary and Pancreatic Surgery, “Victor Babes” University of Medicine and Pharmacy, Eftimie Murgu Square, No. 2, 300041 Timisoara, Romania; octavian.cretu@umft.ro; 13 University Clinic of Physiology, Faculty of Medicine, “Victor Babes” University of Medicine and Pharmacy, Eftimie Murgu Square, No. 2, 300041 Timisoara, Romania; iovin.valentin@umft.ro; 14 X Department of General Surgery, “Victor Babes” University of Medicine and Pharmacy, Eftimie Murgu Square, No. 2, 300041 Timisoara, Romania; 15 Department of Surgery 2, “Pius Brinzeu” Clinical County Emergency Hospital Timisoara, Liviu Rebreanu Boulevard, No. 156, 300723 Timisoara, Romania; 16 Department of Pediatrics I, “Louis Turcanu” Emergency Clinical Hospital for Children, Doctor Iosif Nemoianu Str., No. 2, 300011 Timisoara, Romania; 17 Department of Balneology, Medical Rehabilitation and Rheumatology, “Victor Babes” University of Medicine and Pharmacy, Eftimie Murgu Square, No. 2, 300041 Timisoara, Romania; totorean.alina@umft.ro; 18 Department of Physical and Rehabilitation Medicine, “Pius Brinzeu” Clinical County Emergency Hospital Timişoara, Liviu Rebreanu Boulevard, No. 156, 300723 Timisoara, Romania; 19 Department of Morphopathology, “Pius Brinzeu” Clinical County Emergency Hospital Timisoara, Liviu Rebreanu Boulevard, No. 156, 300723 Timisoara, Romania; 20 Department of Oncology, “Victor Babes” University of Medicine and Pharmacy, Eftimie Murgu Square, No. 2, 300041 Timisoara, Romania; sorin.dema@umft.ro; 21 Department of Radiotherapy, Emergency City Hospital, Victor Babes Str., No. 22, 300595 Timisoara, Romania; 22 Faculty of Medicine, “Vasile Goldis” Western University of Arad, Liviu Rebreanu Str., No. 86, 310045 Arad, Romania; 23 Faculty of Dentistry, “Vasile Goldis” Western University of Arad, Liviu Rebreanu Str., No. 86, 310045 Arad, Romania

**Keywords:** colorectal cancer, primary prevention, nutrition, preventive diet, diet acceptability

## Abstract

**Background/Objectives**: Colorectal cancer is a leading cause of cancer-related death worldwide, with rising incidence in younger adults. Unhealthy diets high in red and processed meat and low in fiber are key modifiable risk factors, highlighting the need for preventive nutritional strategies targeting CRC through dietary interventions. **Methods**: A one-day sample diet for colorectal cancer prevention, consisting of fiber-rich meals excluding red meat and incorporating whole grains, legumes, vegetables, fruits, nuts, and lean protein alternatives (such as fish and poultry), was developed. Its acceptability was assessed in a cross-sectional study using an online questionnaire among healthy Romanian adults aged 18–50, with a total of 395 included participants. **Results**: Of the 395 respondents meeting the inclusion criteria (aged 18–50, no cancer or chronic gastrointestinal disorders), 63.5% were females, predominantly urban (90.1%), and highly educated. Mean age was 32.4 years; mean BMI was 25.07 kg/m^2^. The proposed colorectal cancer-preventive diet was rated as “quite attractive” and “very attractive” by 74.9% of participants. All meals received high ratings, with dinner and the first snack being most favored. Most respondents (77.2%) found the diet satisfying and the satiety level and energy adequate, and 90.4% were willing to adopt it at least a few times per week. Financial accessibility was affirmed by 77.2% of the respondents. However, 61.8% reported difficulty eliminating red meat consumption. Female participants rated the diet significantly more attractive than males did (*p* = 0.041). Willingness to adopt the diet strongly correlated with higher acceptability (*p* < 0.0001), while BMI and education level showed no significant effect. **Conclusions**: The proposed colorectal cancer-preventive diet was well accepted by Romanian adults aged 18–50, with higher receptivity among women and those with higher education; willingness to adopt the diet at least a few days per week was high, especially among those psychologically ready for dietary change, while key barriers included red meat reduction and perceived cost, underscoring the need for gender-sensitive, culturally adapted interventions and further research on long-term adherence and clinical impact.

## 1. Introduction

Colorectal cancer (CRC) remains a significant global public health issue, with high incidence and mortality rates. According to GLOBOCAN 2022 and the American Cancer Society [1,2], CRC is the third most frequently diagnosed cancer and the second leading cause of cancer-related deaths worldwide. Each year, over 1.9 million people are diagnosed, and approximately 935,000 die due to CRC.

A notable and worrying trend is the rise in early-onset colorectal cancer (CRC in individuals under 50), especially in industrialized nations. This trend is linked to modern lifestyle habits such as physical inactivity, obesity, smoking, alcohol consumption, and especially unhealthy diets rich in red and processed meat and low in dietary fiber [3,4,5,6]. These behaviors are considered fundamental modifiable risk factors, with diet playing a central role in disease development.

While developed countries such as the USA, UK, Canada, and Northern European nations show higher CRC incidence, they benefit from established screening programs and early treatment infrastructure [7]. In contrast, low- and middle-income countries are experiencing a sharp increase in CRC incidence due to dietary Westernization, yet often lacking adequate resources for early detection and prevention [8,9].

Nutrition is a key modifiable risk factor for CRC, influencing gut microbiota, epithelial barrier integrity, inflammatory responses, and carcinogen metabolism [10,11,12,13]. One of the most well-documented dietary risk factors is the high intake of red and processed meat. Red meat includes mammalian meats like beef, pork, and lamb, characterized by high myoglobin content. Processed meat refers to products altered through salting, smoking, fermenting, or chemical preservation, such as bacon, sausages, ham, and cold cuts [14].

Numerous studies and meta-analyses confirm a dose-dependent relationship: every 100 g/day of red meat increases CRC risk by 12–18%, and 50 g/day of processed meat increases it by 17% [15,16]. Carcinogenic mechanisms include the formation of heterocyclic amines (HCAs) and polycyclic aromatic hydrocarbons (PAHs) from high-heat cooking, the generation of N-nitroso compounds (NOC) from nitrites and nitrates, and the pro-oxidative effects of heme iron [17,18].

In contrast, high dietary fiber intake from fruits, vegetables, and whole grains has a protective effect. Fiber accelerates intestinal transit, reduces mucosal exposure to carcinogens, and is fermented into short-chain fatty acids (SCFAs) like butyrate, which support epithelial health and have anti-inflammatory and anti-tumor properties [19,20]. Phytochemicals such as polyphenols, flavonoids, and carotenoids modulate gene expression and inhibit pro-oncogenic signaling pathways like NF-κB and Wnt/β-catenin [14].

The gut microbiota plays a crucial intermediary role in the diet–CRC axis. Diet-induced dysbiosis, often from low fiber and high meat intake, promotes pro-inflammatory species like *Fusobacterium nucleatum* and *Bacteroides fragilis*, which impair epithelial integrity and promote tumorigenesis [21,22]. These bacteria produce toxins, induce chronic inflammation, and suppress local immune responses. Furthermore, microbial metabolism can convert dietary compounds into either carcinogens or protective molecules [23,24].

An unhealthy diet can compromise intestinal barrier function, increasing permeability (“leaky gut”), allowing lipopolysaccharides (LPSs) to enter circulation and triggering systemic low-grade inflammation, creating an environment favorable for malignant transformation [25,26]. Dysbiosis also contributes to genomic instability and weakens local antioxidant defenses, accelerating CRC progression [27,28].

Recent clinical trials have shown that dietary interventions, especially Mediterranean or high-fiber diets, can reduce inflammatory markers, polyp burden, and even proto-oncogene expression, thereby lowering the risk of progression from precancerous lesions to invasive CRC [29,30,31]. These findings support the integration of nutritional counseling into preventive medical care and public health multidisciplinary teams.

In conclusion, colorectal cancer is a multifactorial disease with nutrition and lifestyle at its core. Diets high in red and processed meats, low in fiber, and rich in ultra-processed foods contribute to microbial imbalance, chronic inflammation, and carcinogenesis. Conversely, diets rich in fiber and bioactive compounds offer a powerful preventive strategy [32,33,34,35,36]. Addressing these modifiable factors through public health policy, education, and clinical practice is essential to curb the growing CRC burden globally [37,38].

In this paper, we present a one-day sample dietary model designed as a preventive strategy against colorectal cancer, developed in line with established healthy eating guidelines. The model emphasizes the exclusion of red meat and promotes the consumption of fiber-rich foods. The study also investigates how well this dietary approach is received by young adults, using an online questionnaire to assess its acceptability within the local population. Ultimately, the research aims to provide practical recommendations for improving preventive dietary habits in the broader community, based on the insights gathered.

## 2. Materials and Methods

### 2.1. Study Design

This research was conducted as a cross-sectional descriptive study with mixed-method elements, using a self-administered online questionnaire for data collection. The questionnaire included both closed-ended items (quantitative) and open-ended questions (qualitative). It was distributed through social media platforms, within groups focused on preventive nutrition, and via personal networks, to capture diverse viewpoints from the general healthy population. The primary objective was to evaluate the perception and acceptability of a colorectal cancer-preventive dietary model.

### 2.2. Sampling Method and Participant Selection

Participants were recruited through convenience sampling, targeting a diverse pool of individuals from both urban and rural environments in Romania. The recruitment strategy aimed to ensure heterogeneity in terms of educational background, occupational status, and self-reported dietary habits. Eligible participants were healthy adults aged between 18 and 50 years old, without a history of oncological conditions or chronic gastrointestinal diseases.

#### 2.2.1. Inclusion Criteria

- Adults aged over 18 years old.

#### 2.2.2. Exclusion Criteria

- Individuals younger than 18 or older than 50 years;
- History of cancer or chronic gastrointestinal disorders.

### 2.3. Questionnaire Description

The questionnaire was specifically designed for this study and consisted of three sections:

1. Sociodemographic data, including age, sex, education level, and area of residence;
2. Dietary habits and perceptions, assessing the frequency of consumption of various food groups (red meat);
3. Acceptability of the proposed dietary model, evaluated using a series of Likert-scale questions (1–5) covering perceived attractiveness, feasibility, compatibility with personal lifestyle, cost implications, and intention to adopt. Adjacently, open-ended questions were included to allow participants to share observations or suggestions regarding the menu (data not included).

The questionnaire comprised a total of 29 items and required approximately 8–10 min to complete.

The questionnaire was pilot-tested on a sample of 116 participants, of whom 102 were retained after applying eligibility criteria. Internal consistency for the Likert-scale items was assessed using Cronbach’s alpha, which yielded a value of 0.40. Given the multidimensional nature of the constructs being assessed—such as feasibility, motivation, and perceived health benefits—this moderate alpha value was expected. The items were conceptually distinct and not intended to form a single composite scale. As such, results were analyzed and reported at the individual item level using descriptive statistics and correlation analyses, to better reflect the variety of perspectives captured.

Content validation was conducted by a multidisciplinary team of experts, including three specialists in nutrition, eleven clinicians with experience in colorectal cancer, and five specialists in public health, to ensure the relevance and clarity of the instrument. The final version of the questionnaire was distributed in digital format via Google Forms over a one-week period (24 May–31 May 2025).

### 2.4. Statistical Analysis

Collected data were cleaned, organized, and analyzed using Microsoft Excel (version 16.83, Microsoft 365 for macOS), GraphPad Prism (version 10.1.2 for macOS), and R Studio (version 2025.05.0+496). Descriptive statistics were applied to summarize participant characteristics and to report response distributions for each questionnaire item. Categorical variables were presented as frequencies and percentages, while ordinal variables were summarized using medians and interquartile ranges.

To assess differences in responses across participant subgroups, the Mann–Whitney U test was used for non-normally distributed ordinal data. Associations between variables were evaluated using the Spearman rank-order correlation coefficient, appropriate for nonparametric data. A threshold of *p* < 0.05 was considered indicative of statistical significance for all analyses.

Open-ended responses were collected to inform future qualitative analyses; however, they were not included in the current statistical analysis, which focused exclusively on quantitative data from closed-ended items.

### 2.5. Ethics and Consent

Participation in the study was entirely voluntary, and all responses were collected anonymously and treated as strictly confidential. At the beginning of the questionnaire, participants were required to explicitly provide informed consent by selecting a designated agreement checkbox. The questionnaire could not be submitted without this confirmation.

The study was conducted using the Google Forms platform, which allowed only one response per email address to ensure data integrity, while maintaining respondent anonymity. The authors had no access to participants’ email addresses or identifying information; such data remained exclusively within the Google platform’s internal system and was not linked to the research database.

This study qualified for an exemption from formal ethics committee review, as it involved no physical interventions or sensitive personal data, and posed no foreseeable physical, psychological, or legal risks to participants. Prior to data collection, the study protocol was submitted to the Ethics Committee of the Clinical County Emergency Hospital of Arad, Romania, which issued a waiver of ethics approval (Decision No. 92, dated 23 May 2025), in accordance with national guidelines for non-interventional, anonymous survey-based studies. Participation was voluntary, and it could be stopped at any point by abandoning the submission of the questionnaire; all responses were collected anonymously. The study was conducted in accordance with the Declaration of Helsinki and applicable data protection regulations (GDPR). All participants provided explicit informed consent via a mandatory checkbox prior to completing the questionnaire.

## 3. Results

### 3.1. Dietary Model

CRC is strongly influenced by dietary factors. Given the extensive documentation of dietary protective and risk factors, interventions focusing on dietary modifications provide a practical and cost-effective strategy for CRC risk reduction [39]. Based on these principles and the latest insights from nutritional epidemiology and cancer biology, a preventive dietary model for healthy adults was developed. This diet emphasizes plant-rich, fiber-dense, antioxidant-abundant, minimally processed foods, and low red meat, known to modulate CRC risk via inflammation reduction, epigenetic regulation, and microbiota modulation [32,33]. Such a dietary approach not only supports intestinal health but also promotes metabolic balance, ultimately contributing to CRC prevention [16].

**Figure 1:**
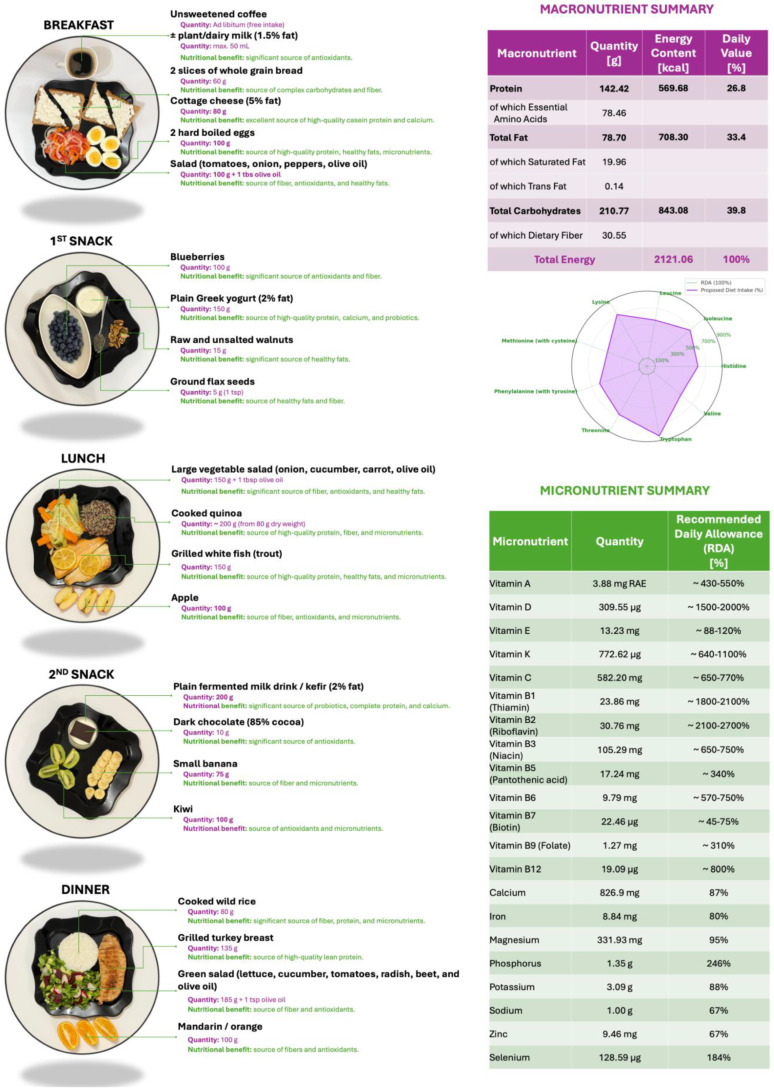
Proposed one-day dietary plan of approximately 2100 kcal in alignment with recommendations for colorectal cancer prevention. On the right, detailed macro- and micronutrient content.

A one-day sample dietary plan providing approximately 2100 kcal/day is proposed, aligned with current recommendations for CRC prevention (Figure 1). This model diet is designed to be nutritionally balanced and protective, and the food quantities are intended to be proportionally adjusted based on the individual’s requirements for total energy expenditure (TEE). TEE values should be calculated using the predictive equations developed by the Committee on Dietary Reference Intakes for Energy of the National Academies of Sciences, Engineering, and Medicine (USA) in collaboration with the National Academy of Sciences and Health Canada, the National Institute of Health (NIH), the U.S. Department of Agriculture (USDA), and the U.S. Food and Drug Administration (FDA) [40]. The predictive equations (Table 1) are stratified by sex and physical activity level (inactive, low active, active, very active) and are applicable to adults (>18 years old).

The menu aligns with EFSA and WHO macro- and micronutrient reference values (Figure 1), ensuring sufficient intake of vitamins A, B-complex, C, D, E, K, and minerals such as calcium, magnesium, and zinc. Structurally, it shows similarities to a Mediterranean dietary model: fiber-rich, low glycemic index, and high in bioactive substances observed to be effective in reducing CRC incidence in diverse populations. The proposed menu integrates whole, minimally processed foods with high fiber, low glycemic load, and anti-inflammatory potential, which target known pathways involved in colorectal carcinogenesis [39,41].

### 3.2. Sample Size Estimation for the Validity of the Questionaire

The required sample size was calculated using a standard formula for estimating proportions, assuming a 95% confidence level (Z = 1.96), a margin of error of ±5% (e = 0.05), and an expected response distribution of *p* = 0.5 to ensure maximum variability. The target population was estimated at approximately 8 million Romanian adults aged 18–50 years [42]. The unadjusted sample size was calculated using Cochran’s Sample Size Formula for Proportions (1953), followed by applying the finite population correction for *N* ≈ 8,000,000:

(1) $$n=\frac{\;\frac{Z^{2}\;\times \;p\;\times \;(1-p)}{e^{2}}}{1+\frac{\;\frac{Z^{2}\;\times \;p\;\times \;(1-p)}{e^{2}}\;-1}{N}}\approx 384$$

Thus, a minimum of 384 participants was considered sufficient to ensure statistical validity and generalizability of the results obtained through the questionnaire as representative for the target Romanian population.

### 3.3. Diet Acceptability Questionaire Analysis

#### 3.3.1. Respondent Demographics

A total of 500 individuals completed the questionnaire. After applying the predefined eligibility criteria—specifically, excluding individuals under the age of 18 or over 50 (67 respondents), and those reporting a history of cancer or chronic gastrointestinal disorders (38 respondents)—a total number of 105 responses were excluded, resulting in a final sample of 395 participants included in the current analysis.

**Figure 2:**
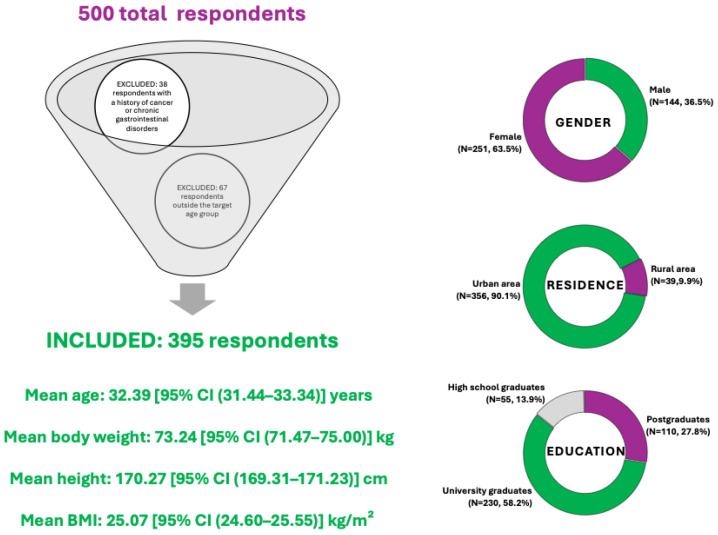
Summary of respondent demographics included in the diet acceptability study for colorectal cancer prevention.

The demographic characteristics of the respondents are summarized in Figure 2. The sample comprised both male (N = 144, 36.5%) and female (N = 251, 63.5%) participants, with varied representation across urban (N = 356, 90.1%) and rural (N = 39, 9.9%) environments. Most participants had a higher education background, with 58.2% holding a university degree (N = 230), 27.8% postgraduate education (N = 110), and 13.9% having completed high school (N = 55). The age range of included participants was 18 to 50 years, with a mean age of 32.39 [95% CI (31.44–33.34)] years. The mean body weight was 73.24 [95% CI (71.47–75.00)] kg, while the average height was 170.27 [95% CI (169.31–171.23)] cm, corresponding to a mean Body Mass Index (BMI) of 25.07 [95% CI (24.60–25.55)] kg/m^2^, which suggests the group is borderline between normal weight and overweight, but statistically, they fall into the overweight range according to WHO classification.

#### 3.3.2. Menu Acceptability Among Respondents

**Figure 3:**
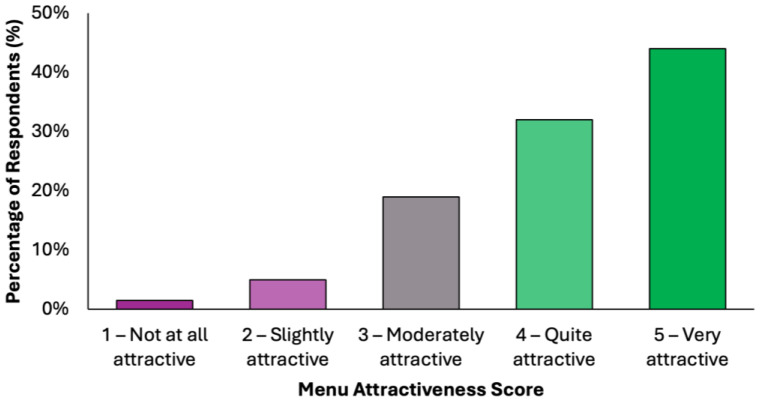
Summary of respondents’ perceived attractiveness of the proposed colorectal cancer-preventive diet, evaluated using a 5-point Likert scale, where 1 indicated “not attractive at all” and 5 indicated “very attractive”.

The perceived attractiveness of the proposed colorectal cancer-preventive diet was evaluated using a 5-point Likert scale, where 1 indicated “not attractive at all” and 5 indicated “very attractive” (Figure 3). Responses revealed a predominantly positive reception: 43.3% of participants (N = 171) rated the diet as “quite attractive”, while an additional 31.6% (N = 125) selected “very attractive”. Combined, these two categories represent 74.9% of the total sample, reflecting a strong overall level of acceptability. A further 20.5% (N = 81) of participants rated the diet as “moderately attractive”, suggesting neutral to slightly positive attitudes, while only a small proportion reported negative views. Specifically, 3.3% (N = 13) found the diet “slightly attractive”, and 1.3% (N = 5) rated it as “not attractive at all”. These results suggest that the proposed menu was generally well received by the surveyed population, with the majority perceiving it as feasible and appealing. The relatively low percentage of negative ratings supports the diet’s potential for broader public health acceptability, particularly in preventive nutrition contexts.

**Figure 4:**
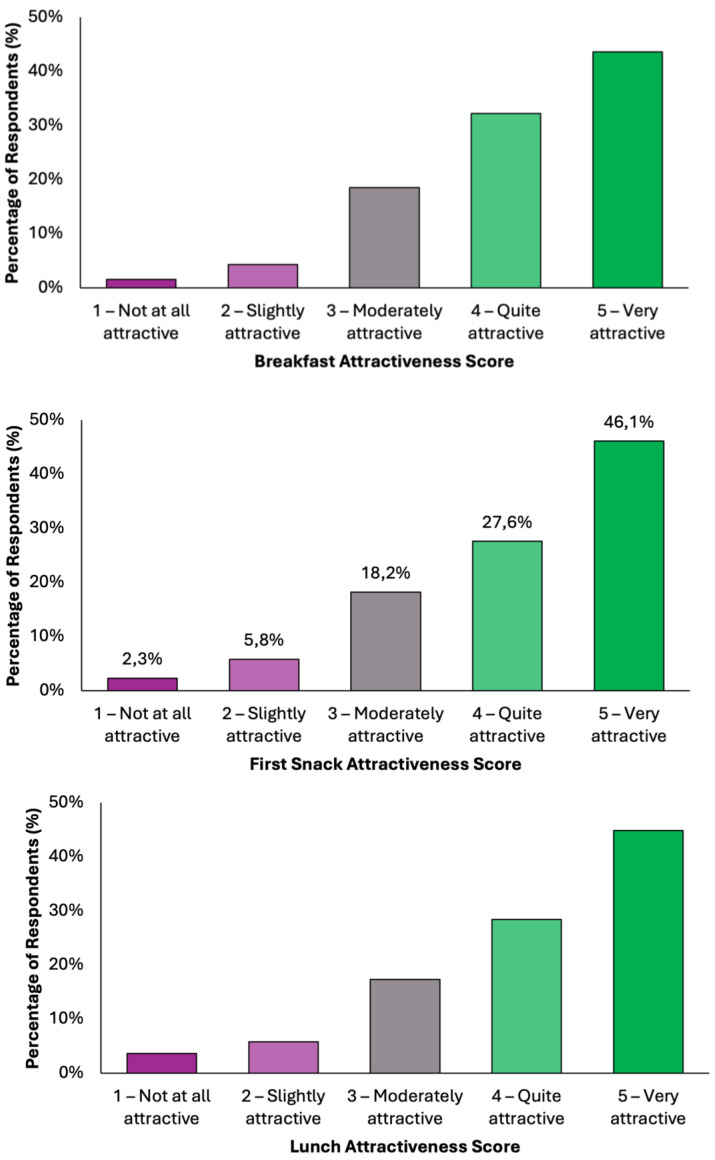

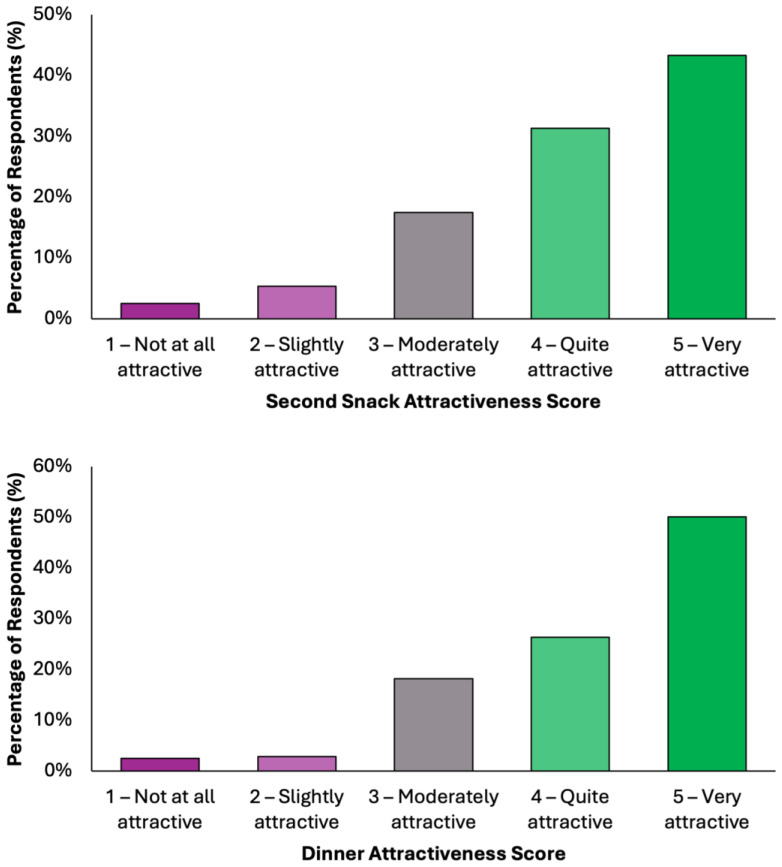
Summary of respondents’ perceived attractiveness of the proposed colorectal cancer-preventive diet, evaluated for every meal using a 5-point Likert scale, where 1 indicated “not attractive at all” and 5 indicated “very attractive”.

Overall, the data indicate a high level of acceptability across all meals, with most responses falling within the upper two categories of attractiveness (Figure 4). Dinner emerged as the most favorably rated meal, with 50.1% (N = 198) of respondents describing it as “very attractive”, followed by 26.3% (N = 104) who found it “quite attractive”. Together, over 76% of participants viewed Dinner as highly appealing. Similarly, Snack 1 received a very positive reception, with 46.1% (N = 182) rating it as “very attractive” and 27.6% (N = 109) as “quite attractive”. Lunch was also well received, with 45.1% (N = 178) finding it “very attractive” and 28.4% (N = 112) “quite attractive”. Breakfast and Snack 2 also performed strongly, each receiving “very attractive” ratings from 43.5% (N = 172) and 43.3% (N = 171) of respondents, respectively. Across all meals, the majority of responses (70–77%) fell into the top two categories, indicating a broadly positive perception. Negative responses were minimal, ranging between 5.8 and 9.3%, depending on the meal. These findings demonstrate a consistent pattern of favorable evaluation, suggesting that the proposed dietary model is not only nutritionally aligned with colorectal cancer prevention goals but also broadly acceptable in terms of taste and meal appeal to the general adult population.

**Figure 5:**
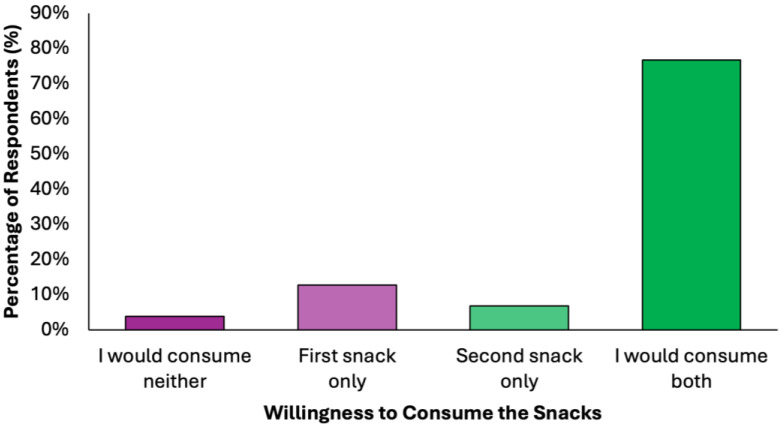
Summary of respondents perceived willingness to consume the proposed snacks of the colorectal cancer-preventive diet.

More than three quarters of respondents (N = 303, 76.7%) expressed willingness to consume both snacks, indicating high acceptability (Figure 5). The first snack was slightly more preferred than the second snack among those who selected only one option (N = 50, 12.7% vs. N = 27, 6.8%, respectively). The rejection rate was minimal (N = 15, 3.8%), further supporting the feasibility of integrating these snacks into preventive dietary models.

**Figure 6:**
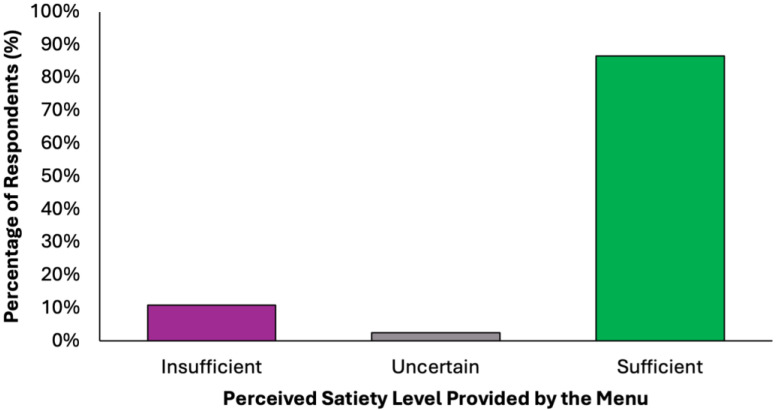

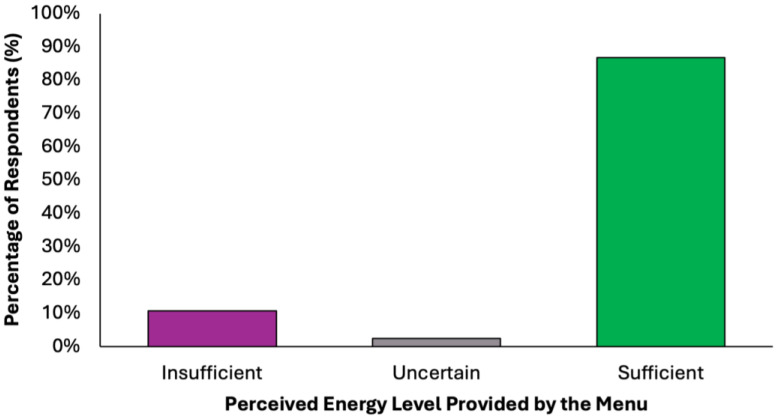
Summary of respondents’ perceived adequacy of satiety and energy provided by the proposed colorectal cancer prevention diet.

The perceived adequacy of satiety and energy provided by the proposed colorectal cancer prevention diet was assessed using a closed-ended question with five response options (Figure 6). A substantial majority of respondents (77.2%, N = 305) agreed that the menu was sufficient both in terms of satiety and daily energy needs, suggesting that the diet was generally perceived as balanced and nutritionally complete. Two subgroups of partial agreement were identified: 9.6% (N = 38) of respondents reported that the diet provided adequate energy but lacked satiety, while 9.4% (N = 37) found it satisfying in terms of fullness but insufficient in energy content. These nuanced responses suggest that while most participants perceived the menu positively, a notable minority highlighted areas where macronutrient balance or caloric density might require individual adjustments. A small percentage of participants expressed uncertainty (2.5%, N = 10) or a clearly negative assessment (1.3%, N = 5), stating that the menu was insufficient in both energy and satiety. These results confirm a very high level of perceived satiety (N = 380, 96.2%) and strong perceived energy adequacy (N = 348, 88.1%), with a minority of undecided or negative responses in both domains.

**Figure 7:**
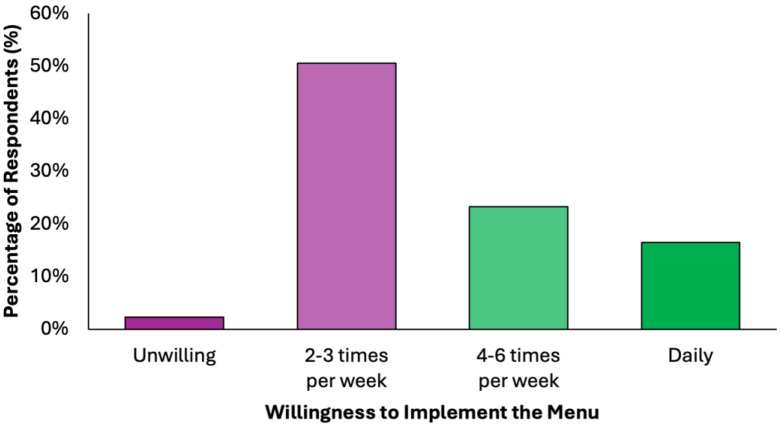
Summary of respondents’ perceived readiness for partial or full integration of the proposed colorectal cancer-preventive diet in the routine.

Half of the respondents (50.6%) indicated willingness to implement the proposed dietary model 2–3 times per week, while another 23.3% would adopt it for 4–6 days per week, and 16.5% expressed readiness for daily use (Figure 7). Only 2.3% rejected the idea entirely. This indicates a broad readiness for partial or full integration, supporting the practicality of the proposed preventive menu in real-world settings.

**Figure 8:**
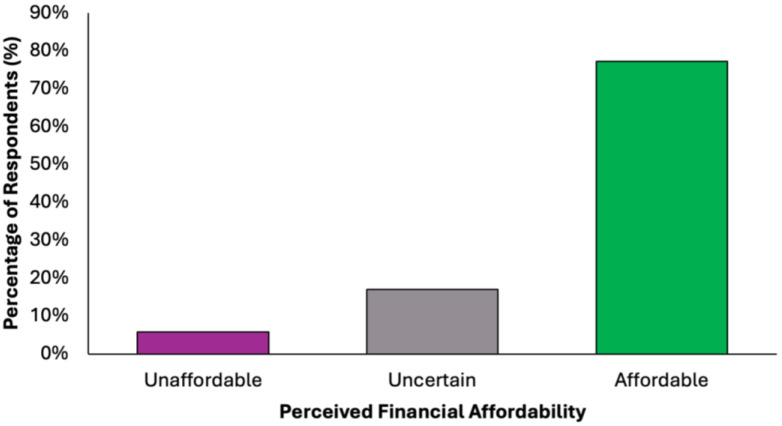
Summary of respondents’ perceived financial accessibility of the proposed colorectal cancer-preventive diet.

The majority of participants (N = 305, 77.2%) perceived the proposed diet as financially accessible, while a small proportion (N = 23, 5.8%) considered it unaffordable (Figure 8). A notable minority (N = 67, 17.0%) were undecided, suggesting variability in cost perception based on individual financial circumstances. These findings support the potential for broad implementation of the dietary model across diverse socioeconomic groups.

**Figure 9:**
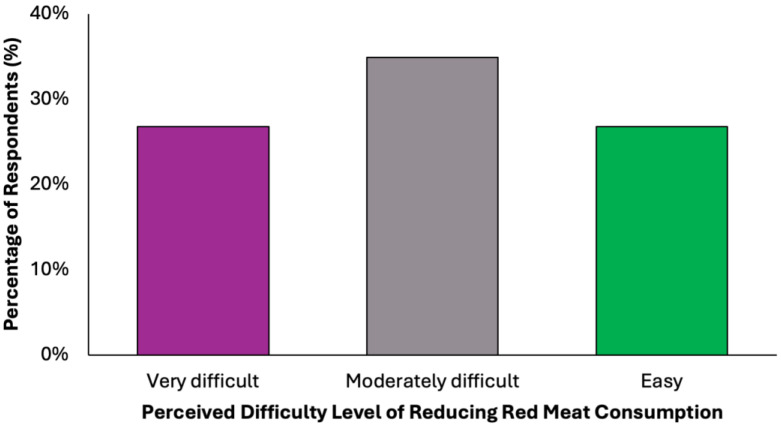
Summary of respondent willingness to eliminate red meat for a colorectal cancer-preventive diet.

In evaluating the acceptability of the proposed dietary model, a key limiting factor appears to be participants’ readiness to eliminate red meat, a well-established dietary risk factor for colorectal cancer (Figure 9). Only 38.2% (N = 151) of respondents stated they would not find it difficult to give up red meat. In contrast, the remaining 61.8% expressed varying degrees of resistance: 34.9% (N = 138) reported partial difficulty, while 26.8% (N = 106) found the prospect clearly difficult. These findings reveal a notable level of reluctance toward one of the most impactful dietary modifications recommended for colorectal cancer prevention.

### 3.4. Factors Influencing Diet Acceptability

**Figure 10:**
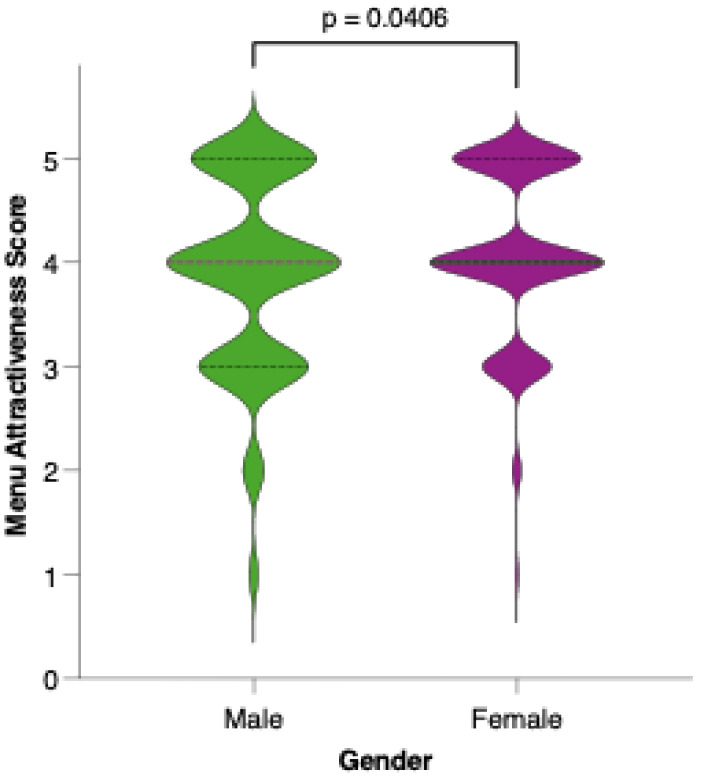
Perceived attractiveness of the proposed colorectal cancer-preventive diet by gender. Violin plots show the distribution of attractiveness ratings (1–5 Likert scale) for female (red) and male (blue) participants. The bold horizontal line within each violin indicates the group mean. Note: Statistically significant difference in Mann–Whitney U test as indicated by the significance line above the plot.

Female participants generally rated the proposed menu as more attractive than their male counterparts did. As shown in Figure 10, the distribution of attractiveness scores among females was shifted toward higher values, with a greater proportion assigning scores of 4 or 5 on the Likert scale. Statistical comparison using the Mann–Whitney U test confirmed that this difference was significant (*p* = 0.0012), suggesting that gender may play a role in the perceived appeal and acceptability of the colorectal cancer-preventive dietary model.

**Figure 11:**
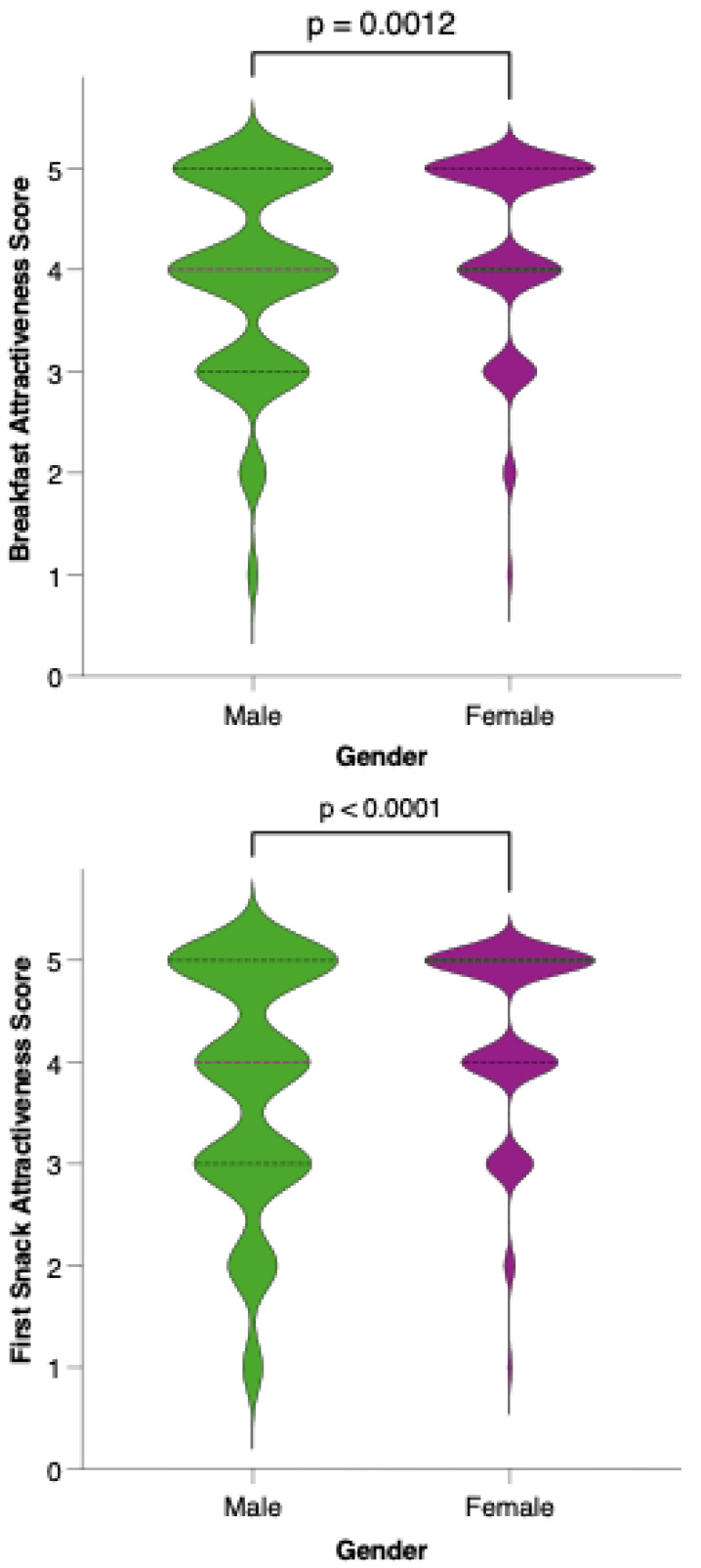

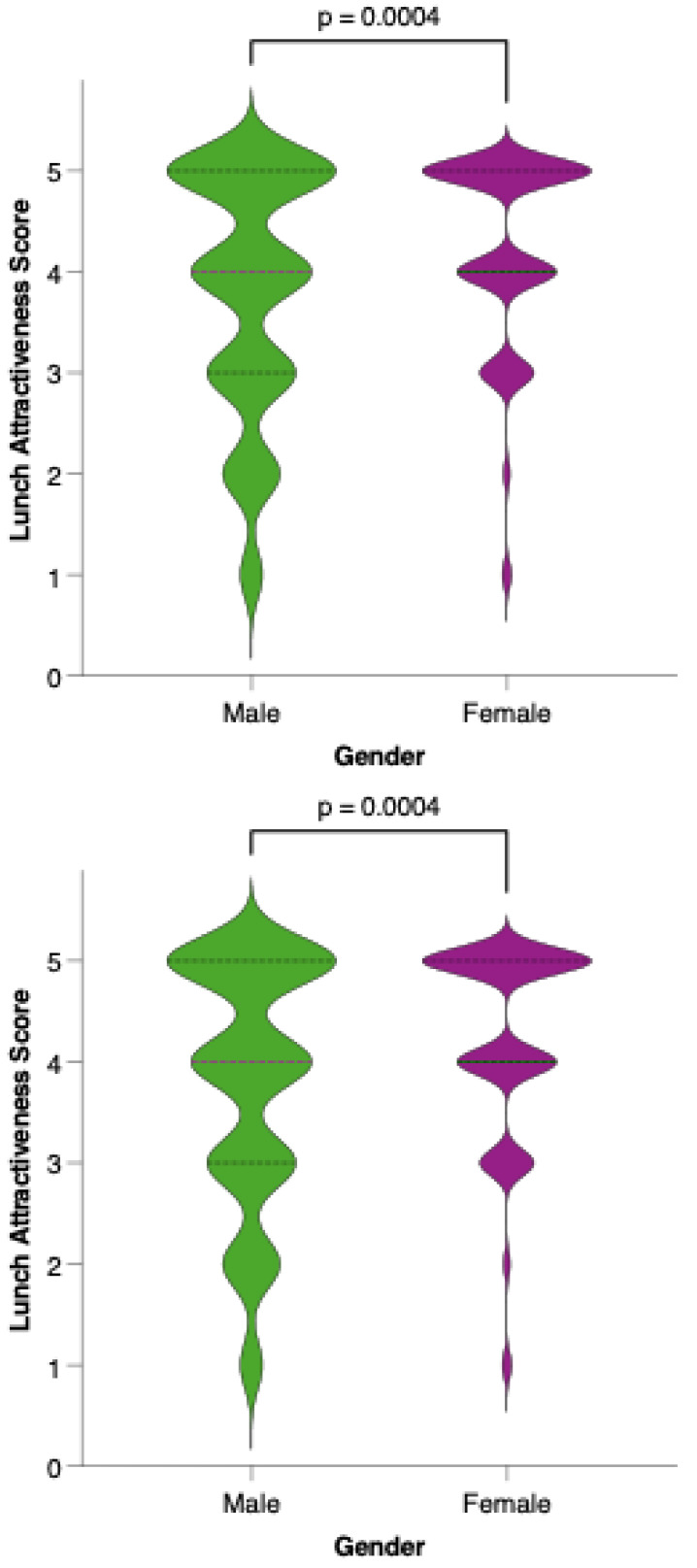

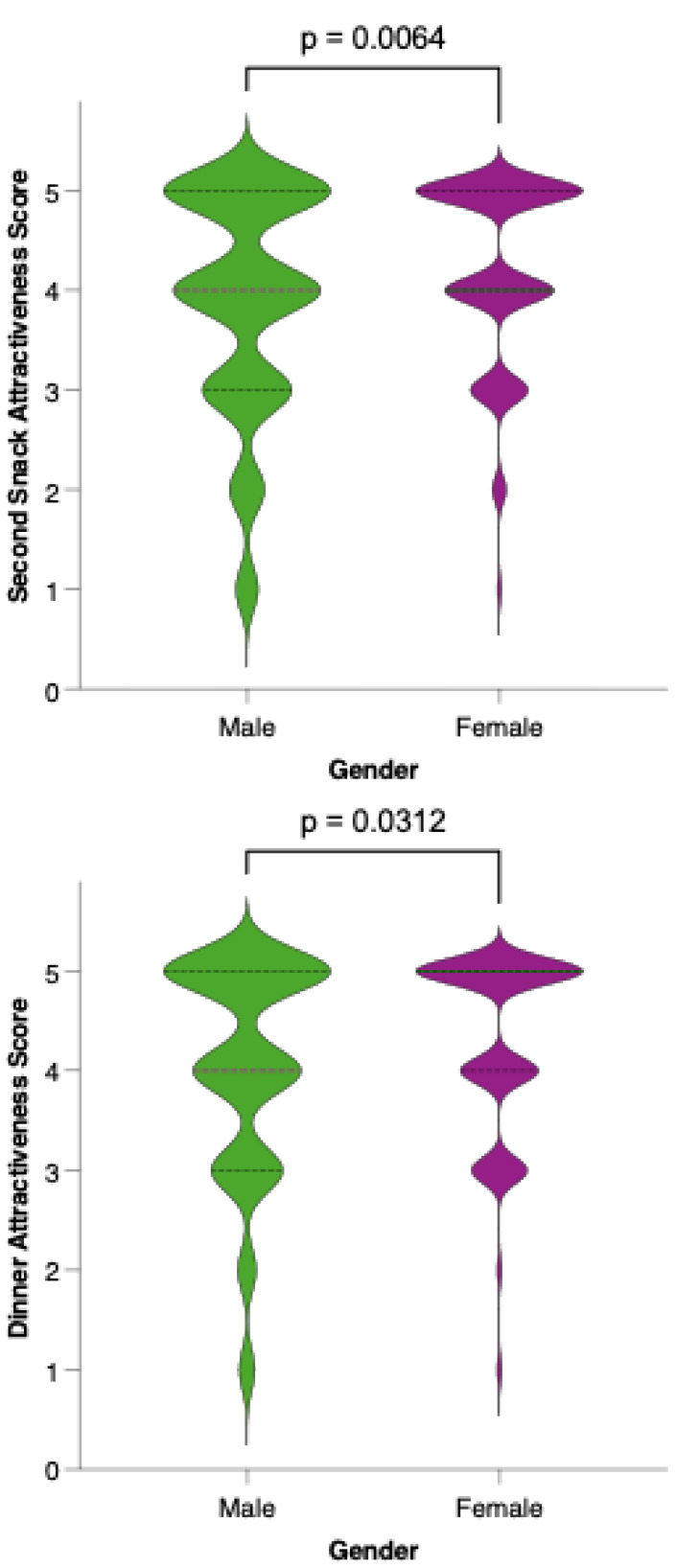
Perceived attractiveness of each proposed meal in the colorectal cancer-preventive diet by gender. Violin plots show the distribution of attractiveness ratings (1–5 Likert scale) for female (red) and male (blue) participants. The bold horizontal line within each violin indicates the group mean. Note: Statistically significant difference in Mann–Whitney U test as indicated by the asterisk and significance line above the plot.

Analysis of meal-specific attractiveness ratings revealed notable differences in perception between female and male participants for all proposed menu items (Figure 11). For each meal—breakfast, first snack, lunch, second snack, and dinner—female respondents consistently rated the attractiveness of the options higher than their male counterparts. None of the meals reached a level of equal acceptability across genders, as reflected by statistically significant differences (*p* < 0.05) in the Mann–Whitney U tests for all comparisons. This pattern suggests that, while the dietary model is generally well received, its universal appeal is limited by gender-based preferences.

**Figure 12:**
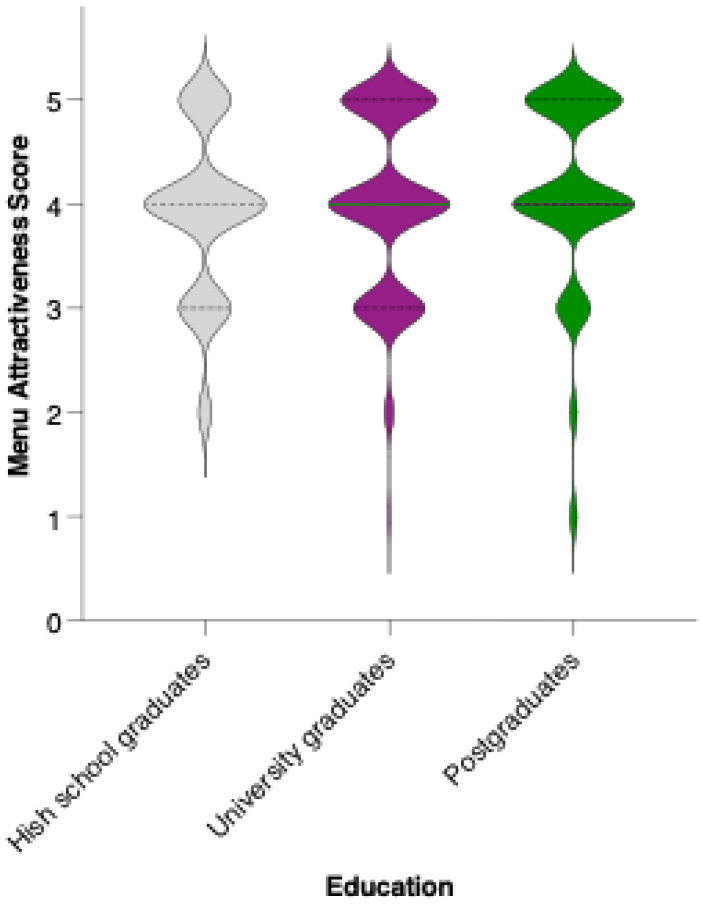
Perceived attractiveness of the proposed colorectal cancer-preventive diet by educational level. Violin plots show the distribution of attractiveness ratings (1–5 Likert scale) for high school graduates (blue), university graduates (grey), and postgraduates (white). The bold horizontal line within each violin indicates the group mean. Note: Mann–Whitney U test revealed no statistically significant differences between groups (n.s.).

While there is a visible trend toward higher average scores among university and postgraduate respondents compared to those with a high school education, statistical analysis revealed that these differences did not reach significance in pairwise comparisons (Mann–Whitney U test, ns) (Figure 12). The mean attractiveness scores for all groups fell within the positive range, supporting the menu’s general acceptability across educational backgrounds. However, the trend suggests that individuals with higher educational attainment may be somewhat more receptive to preventive dietary interventions.

Analysis of the relationship between BMI and the perceived attractiveness of the proposed colorectal cancer-preventive diet, using both linear regression and nonparametric Spearman rank correlation, revealed no statistically significant association in this sample. The correlation coefficients for both methods were nearly zero, indicating an absence of linear or monotonic trends. In practical terms, this suggests that respondents’ perceptions of the diet’s appeal were not meaningfully influenced by their body weight status.

**Figure 13:**
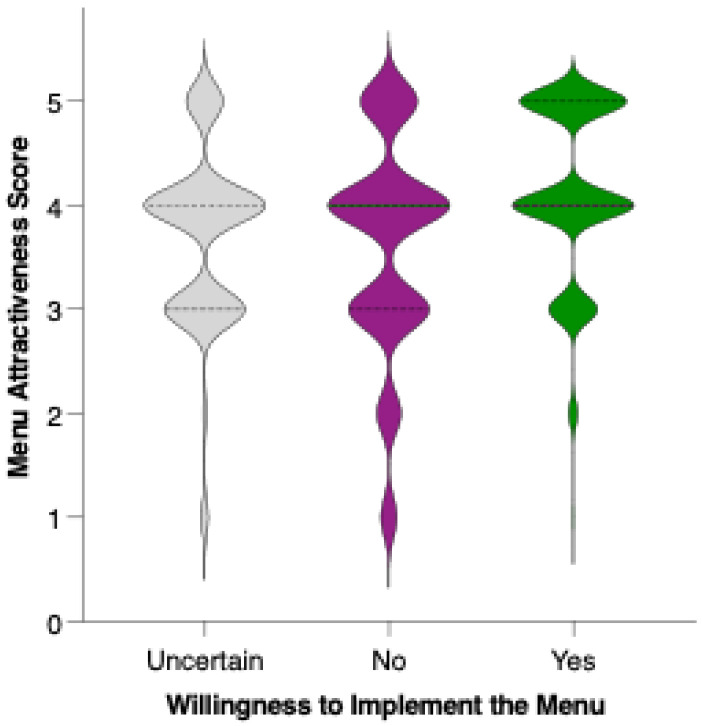
Perceived attractiveness of the proposed colorectal cancer-preventive diet by willingness to replace the usual menu. Violin plots show the distribution of attractiveness ratings (1–5 Likert scale). The bold horizontal line within each violin indicates the group mean. Note: Statistically significant difference in Mann–Whitney U test as indicated by the asterisk and significance line above the plot.

Respondents who indicated a clear willingness to replace their usual menu (“Yes”) rated the proposed menu significantly higher in acceptability compared to both those who were uncertain (“I don’t know”; *p* = 0.0021, **) and those unwilling to make the substitution (“No”; *p* < 0.0001, ****). The mean acceptability score was lowest among respondents who were not willing to replace their usual menu, intermediate for those who were undecided, and highest among those expressing willingness to adopt the new diet (Figure 13). These differences were statistically significant for both “No” vs. “Yes” and “I don’t know” vs. “Yes” comparisons, indicating a clear trend: greater openness to dietary change is associated with more favorable perceptions of the preventive menu. The substantial gap in scores between the “No” and “Yes” groups highlights that acceptability is not only influenced by the nutritional or sensory qualities of the menu but also by participants’ readiness to make substantial dietary changes.

## 4. Discussion

### 4.1. Dietary Model

The menu’s vitamin composition strongly supports its role in colorectal cancer prevention [43]. With potent antioxidant, immunomodulatory, and epigenetic effects, these vitamins help mitigate pathways involved in CRC initiation and progression. The nutrient synergy between fat-soluble and water-soluble vitamins further enhances metabolic resilience and gut mucosal defense [44].

The protein-rich items (Figure 1) provide all essential amino acids and contribute to satiety, muscle maintenance, and metabolic balance [45]. Eggs are a source of choline and selenium, both associated with improved mucosal health and immune response [46,47]. While early studies debated their cholesterol content, recent analyses confirm no association with increased CRC risk when consumed in moderation [48]. Dairy products, particularly fermented ones like kefir and yogurt, offer lactic acid bacteria that enhance colonic epithelial barrier function and reduce local inflammation [49,50]. Greek yogurt contains higher protein and calcium concentration, promoting DNA repair and apoptosis regulation. Trout is rich in omega-3 fatty acids, particularly EPA and DHA, which are known to reduce cyclooxygenase-2 (COX-2) expression and suppress tumor growth [51,52]. Turkey breast is a lean protein source low in saturated fats and free of heme iron, a known pro-oxidant linked with CRC [53].

Whole grains and seeds contribute significantly to dietary fiber, resistant starch, lignans, and polyphenols. Regular intake of whole grains is consistently associated with lower CRC risk, with multiple meta-analyses indicating a 17% reduction per 90 g/day increment (RR = 0.83, 95% CI: 0.78–0.88) [54,55,56]. Flax seeds provide alpha-linolenic acid and secoisolariciresinol diglucoside (SDG), a lignan with phytoestrogenic and antioxidant activity, implicated in suppressing colorectal tumorigenesis [57,58]. Quinoa, a pseudo-cereal, offers a complete amino acid profile and is rich in saponins, phenolic acids, and flavonoids with demonstrated antiproliferative effects [59].

Fruits are central to antioxidant and fiber intake in CRC-preventive diets. Apples are rich in polyphenols and pectin, which may inhibit colon carcinogenesis by reducing oxidative stress and modulating detoxification pathways [60]. A meta-analysis found a 25% reduction in CRC risk among regular apple consumers (OR = 0.75, 95% CI: 0.67–0.84) [61,62,63]. Blueberries are high in anthocyanins and have demonstrated antiproliferative and anti-inflammatory effects [64]. Kiwi and citrus fruits such as mandarins offer 13% (RR = 0.87) and 9% (RR = 0.91) risk reductions for CRC, respectively [65]. Bananas, especially semi-ripe, provide resistant starch that enhances SCFA production and microbial diversity, with some benefit noted in individuals with Lynch syndrome [66]. Regular banana intake was associated with an OR = 0.72 for CRC [67], although data are heterogeneous.

Vegetables contribute fiber, vitamins, and phytochemicals critical for CRC prevention. Tomatoes provide lycopene, a potent antioxidant [68]. Allium vegetables such as onions contain organosulfur compounds that promote phase II enzyme activity and reduce mutagenesis [69,70]. Carrots supply beta-carotene and luteolin, linked with lower oxidative stress and improved immune response [71]. Leafy greens and root vegetables like beet and radish are high in folate, potassium, and nitrates, which support mucosal defense [32].

Walnuts contain PUFA, ellagitannins, and phytosterols with demonstrated CRC-preventive properties through microbiota modulation and inflammatory pathway regulation [72]. Olive oil provides oleic acid and polyphenols such as hydroxytyrosol, known to modulate oncogenic signaling and reduce CRC risk [73].

Coffee provides phenolic acids and diterpenes (cafestol, kahweol) which may inhibit tumor growth and support antioxidant defenses [74]. Moderate coffee intake is not linked with increased CRC risk and may be protective [75,76]. Dark chocolate, high in flavanols, modulates inflammation and insulin sensitivity, factors involved in CRC progression [77].

The menu aligns with EFSA and WHO nutrient reference values, ensuring sufficient intake of vitamins A, C, D, E, K, B-complex, and minerals like calcium, magnesium, and zinc [78,79,80,81]. Structurally, it reflects a Mediterranean dietary model—rich in fiber, low in glycemic index, and high in bioactives—which has been proven effective in reducing CRC incidence in diverse populations [33,82].

While the associations between specific foods and reduced colorectal cancer risk are supported by numerous epidemiological and mechanistic studies, causality cannot always be established. Many findings are based on observational data subject to residual confounding and dietary reporting bias. Moreover, effect sizes vary by population genetics, baseline microbiota composition, and dietary patterns. Therefore, while this menu aligns with current preventive guidelines and biologically plausible mechanisms, clinical trials are needed to confirm its long-term protective effects across diverse populations.

### 4.2. Diet Acceptability

The final sample included 395 participants, exceeding the minimum required sample size of 384 respondents, as determined by the sample size calculation based on Cochran’s formula adjusted for a finite population of approximately 8 million Romanian adults aged 18–50 years [42]. This calculation assumed a 95% confidence level, a margin of error of ±5%, and a maximum variability assumption (*p* = 0.5) to ensure the broadest generalizability. Therefore, the achieved sample size meets the threshold for statistical validity and supports the representativeness and reliability of the findings. The exclusion of 105 respondents—due to age ineligibility (N = 67) or self-reported history of oncological or chronic gastrointestinal conditions (N = 38)—was consistent with the study’s inclusion criteria and further contributed to the internal validity of the analysis by ensuring a homogeneous population of healthy adults within the target age range.

The demographic profile of the study sample offers both strengths and limitations for interpreting the results. The predominance of female participants (63.5%) is consistent with trends in voluntary health research, where women typically show greater involvement in preventive behaviors and study participation [83]. Nonetheless, this gender imbalance may constrain generalizability, as colorectal cancer (CRC) incidence and mortality are higher among men [84]. This under-representation suggests a need for gender-sensitive strategies to better engage male populations at elevated risk.

Educationally, the sample was highly qualified, with over 85% holding university or postgraduate degrees. While this likely facilitated the high receptiveness to preventive dietary practices observed, it may not accurately reflect perspectives of individuals with lower educational attainment, who often face greater challenges in adopting dietary changes and cancer-preventive behaviors [39]. Similarly, the overwhelming representation of urban residents (90.1%) mirrors national migration trends among younger and middle-aged adults in Romania [42,85], yet may limit applicability of the results to rural populations with different levels of access to health resources and dietary options.

Conversely, the mean age (32.4 years) and BMI (25.07 kg/m^2^) are well aligned with national profiles for CRC prevention targets, underscoring the relevance of including adults aged 18–50—a critical window for establishing long-term health behaviors with potential to reduce cancer risk. Overall, while the sample is well suited for investigating prevention among health-conscious, at-risk adults, interpretations should account for its gender, educational, and urban/rural composition.

The results indicate a high overall acceptability of the proposed dietary model, with the majority of participants rating both the full menu and its individual meals as attractive or very attractive. Given the role of diet as a key modifiable risk factor in colorectal cancer (CRC) prevention [39,86], these findings are encouraging. They align with international evidence demonstrating that well-structured, fiber-dense, nutrient-rich diets can be both appealing and feasible in everyday settings [16,32]. Approximately 75% of respondents evaluated the full menu positively, with consistent approval across most meals. Dinner emerged as the most favorably rated, consistent with prior findings suggesting evening meals tend to score higher in acceptability, potentially due to increased variety, larger portions, or cultural preferences [39,87]. Breakfast and snack items, while still well received, showed greater variability in preference and more pronounced gender differences, indicating areas for targeted refinement. The high acceptance of both snacks (over 75% willing to consume both) and the menu’s ability to provide perceived satiety and adequate energy for most participants (over 88% reporting adequacy in both domains) support the feasibility of implementing such a model in preventive nutrition.

Importantly, most respondents were willing to adopt the menu at least several days per week, if not daily, indicating realistic possibilities for integration into contemporary dietary patterns. These results are in line with international reports on the acceptability and uptake of plant-based or Mediterranean-style diets in cancer prevention [88,89].

Despite these favorable outcomes, certain barriers to widespread adoption were identified. While most participants viewed the menu as financially accessible, a significant minority expressed uncertainty or concern about affordability. This highlights the need for economic considerations in dietary interventions and potential support mechanisms for lower-income populations [90,91]. The most prominent behavioral barrier was resistance to reducing red meat consumption. Romania has one of the highest per capita red meat consumption rates in Europe [92,93]. Thus, expectedly, in this study, only 38.2% of participants indicated no difficulty with meat elimination, while the rest reported varying degrees of challenge. This reflects global patterns showing that red meat is deeply embedded in dietary habits and that adherence to cancer-preventive guidelines on meat reduction remains challenging in many populations [39,87]. To overcome this, culturally sensitive strategies and gradual substitution approaches may be necessary [94].

Notably, the study revealed consistent gender differences in the acceptability of both the overall menu and individual meals, with female participants rating all items significantly higher than males. No meal received equivalent favorability across genders, with the largest disparities being observed for breakfast and snacks. These findings highlight the importance of gender-tailored dietary interventions to improve both appeal and public health outcomes, particularly given that men are less likely to adopt plant-forward diets and face a higher risk of colorectal cancer [84,95,96]. Specifically, the second snack (kefir, banana, and dark chocolate) and breakfast emerged as components requiring targeted adjustments, such as alternative ingredients or presentation techniques, to better meet male preferences and enhance acceptability. In contrast, dinner exhibited the smallest gender gap alongside the highest overall attractiveness, suggesting its potential as a model for developing gender-neutral, widely accepted menu options. Tailoring preventive diet menus to address male tastes and behavioral barriers is therefore critical to increasing dietary adherence and ultimately reducing colorectal cancer burden in this high-risk population [39].

This result is consistent with the existing literature, which suggests that dietary preferences and the acceptability of preventive menus are more strongly influenced by cultural, educational, and psychosocial factors than by anthropometric variables such as BMI [97,98]. Although individuals with higher BMI might be expected to show increased openness to dietary interventions, the current data indicate that BMI alone does not predict menu appeal. Conversely, those with lower BMI—often already engaged in health-conscious behaviors—may be more inclined to favor such preventive models due to pre-existing positive attitudes toward nutrition and lifestyle. Taken together, these findings underscore the complex interplay of multiple determinants, beyond BMI alone, that influence receptiveness to preventive dietary models.

The present study indicates that female participants consistently perceived the proposed preventive menu and its constituent meals as more attractive than their male counterparts. These gender-based differences were statistically significant across all menu components as well as in the overall rating, with women’s responses clustering toward the upper end of the Likert scale. These findings are consistent with the previous literature demonstrating a greater openness among women to health-promoting dietary interventions, particularly those centered on plant-based and preventive models [95,96]. Such disparities may be partially explained by sociocultural factors, including entrenched associations between meat consumption and masculine identity, as well as gender differences in health-related motivation and behavior [99,100]. Given that men bear a disproportionately higher global burden of colorectal cancer [101], these findings highlight the critical importance of designing gender-sensitive nutritional strategies that address specific perceptual and behavioral barriers to facilitate greater adoption of preventive dietary practices among male populations.

Although participants with university or postgraduate education rated the menu slightly higher than those with only secondary education, these differences were not statistically significant. Nevertheless, the trend supports broader evidence linking higher educational attainment with increased nutritional awareness and proactive health behaviors [97,102]. Future interventions may benefit from educational customization to enhance relevance and uptake across diverse literacy levels.

Interestingly, BMI was not significantly correlated with menu attractiveness in either linear or nonparametric analyses. This supports the notion that dietary receptiveness is driven more by psychosocial and cultural dynamics than by weight status alone [98,103]. The absence of a clear association suggests that motivation and readiness for change may be more critical than BMI in shaping intervention success.

The most salient predictor of menu acceptability was respondents’ stated willingness to adopt the proposed dietary model. Those ready to replace their usual meals with the preventive menu gave significantly higher ratings than those expressing hesitation or resistance. This finding reinforces the central role of motivational readiness in nutritional interventions, as emphasized by behavioral change theories such as the Transtheoretical Model [104,105]. Aligning intervention content with individuals’ stage of change and addressing perceived dietary and cultural barriers appear essential for improving adherence and public health impact.

### 4.3. Limitations

Several limitations must be acknowledged when interpreting the findings of this study. Notably, the sample composition was skewed toward females (63.5%), individuals with high educational attainment (over 85% university or postgraduate degrees), and urban residents (90.1%), which may constrain the generalizability of results to the wider Romanian population. This demographic profile, typical of voluntary online health surveys, is known to be associated with heightened dietary awareness and engagement in preventive behaviors, potentially biasing estimates of menu acceptability upward [106]. Moreover, the cross-sectional design, based on self-reported data collected via online questionnaires, raises concerns regarding reporting bias and social desirability effects, as participants may overstate favorable perceptions or under-report barriers related to the preventive diet [107]. The assessment of hypothetical willingness to adopt the menu, rather than observed dietary behavior, limits insight into real-world adherence and long-term acceptability.

Additional limitations include the evaluation of the dietary model over only one day rather than assessing long-term adherence and potential financial or behavioral barriers, such as difficulty reducing red meat intake, which may affect the feasibility and sustainability of the proposed dietary changes. Regarding the proposed preventive menu, the cultural context of Romania, within which the menu and questionnaire were developed and tested, may limit extrapolation to populations with distinct culinary traditions, ingredient availability, or health beliefs. Furthermore, the need for culturally tailored adjustments to enhance real-world feasibility across diverse socioeconomic groups should be considered to ensure the broader applicability and effectiveness of the dietary model.

### 4.4. Implications for Practice and Policy

The observed discrepancies in diet acceptability across gender, education level, and motivational readiness underscore the necessity of adopting a personalized, multilevel approach in both public health practice and policy formulation. Policies aiming to reduce colorectal cancer incidence through dietary interventions should not only address nutritional content but also integrate behavioral science principles to enhance adherence.

Future research should address long-term adherence to the proposed dietary model, beyond the evaluation of a single-day menu, to determine sustained feasibility, acceptability, and health impact. It should evaluate clinical outcomes of diet adoption in prospective interventional studies, including large cohort cancer risk reduction. It should explore behavioral barriers, particularly reluctance to red meat reduced intake, by integrating tailored educational, motivational, and gradual substitution strategies in dietary interventions. This could lead to the development of culturally resonant dietary adaptations to ensure the model is acceptable and practical in diverse regional and cultural contexts within Romania and internationally. Furthermore, investigating gender-specific preferences and barriers should be investigated to design targeted interventions, particularly engaging male populations at higher CRC risk. Last, but not least, cost-effectiveness analyses should be conducted to assess financial feasibility across socioeconomic strata and identify supportive policies or subsidies needed for widespread adoption.

Moreover, as willingness to adopt preventive diets was closely associated with individual motivation rather than anthropometric indicators such as BMI, future programs should prioritize behavioral assessment tools in early intervention planning to tailor support accordingly.

Integrating these research directions into national and regional cancer prevention frameworks could significantly improve the reach, equity, and efficacy of dietary prevention strategies against colorectal cancer.

## 5. Conclusions

This study highlights a generally high level of acceptability for the colorectal cancer-preventive dietary model among Romanian adults aged 18–50. Most participants rated both the overall menu and its individual meals as attractive, with acceptability influenced primarily by gender and education level. Female respondents and those with higher educational attainment were more receptive, while notable gender differences emerged especially for breakfast and snack items. Importantly, individuals expressing a clear willingness to adopt the proposed diet rated it significantly higher, emphasizing the central role of psychological readiness in dietary change. Barriers to adoption were relatively limited, with the main concerns involving the reduction of red meat and perceived financial burden. BMI was not associated with menu attractiveness, suggesting that sociocultural, educational, and psychosocial factors are more predictive of dietary receptivity than anthropometric indicators. Overall, the findings support the feasibility of introducing a fiber-rich, nutrient-rich diet as a preventive strategy for colorectal cancer in younger and middle-aged adults. However, the success of such interventions will depend on culturally adapted, gender-sensitive approaches, particularly to improve engagement among men and populations with lower education or from rural areas. Future studies should explore long-term adherence and clinical effectiveness of this dietary model.

## Figures and Tables

**Table 1:** Predictive equations of individual total energy expenditure (TEE) stratified by sex and physical activity level (inactive, low active, active, very active). Equations adapted from the Consensus Study Report of the Committee on Dietary Reference Intakes for Energy [40].

Physical Activity Level (PAL)	Predictive Equations of Individual Total Energy Expenditure (TEE) in Adults (>18 Years Old)	
	Female	Male
Inactive	**584.90** – (**7.01** × age) + (**5.72** × height) + (**11.71** × weight)	**753.07** – (**10.83** × age) + (**6.50** × height) + (**14.10** × weight)
Low active	**575.77** – (**7.01** × age) + (**6.60** × height) + (**12.14** × weight)	**581.47** – (**10.83** × age) + (**8.30** × height) + (**14.94** × weight)
Active	**710.25** – (**7.01** × age) + (**6.54** × height) + (**12.34** × weight)	**1004.82** – (**10.83** × age) + (**6.52** × height) + (**15.91** × weight)
Very active	**511.83** – (**7.01** × age) + (**9.07** × height) + (**12.56** × weight)	– **517.88** – (**10.83** × age) + (**15.61** × height) + (**19.11** × weight)

Note: Total energy expenditure (TEE)—unit of measurement: kcal; Age—unit of measurement: years; Height—unit of measurement: cm; Weight—unit of measurement: kg.

## Data Availability

The original contributions presented in this study are included in the article. Further inquiries can be directed to the corresponding authors.
